# Patients’ Satisfaction with HIV Care Providers in Public Health Facilities in Lusaka: A Study of Patients who were Lost-to-Follow-Up from HIV Care and Treatment

**DOI:** 10.1007/s10461-019-02712-4

**Published:** 2019-10-31

**Authors:** Njekwa Mukamba, Obvious N. Chilyabanyama, Laura K. Beres, Sandra Simbeza, Kombatende Sikombe, Nancy Padian, Charles Holmes, Izukanji Sikazwe, Elvin Geng, Sheree R. Schwartz

**Affiliations:** 1 Centre for Infectious Disease Research in Zambia, Lusaka, Zambia; 2 Department of International Health, Johns Hopkins School of Public Health, Baltimore, MD USA; 3 Division of Epidemiology, University of California, Berkeley, Berkeley, CA USA; 4 Division of Infectious Diseases, Johns Hopkins University School of Medicine, Baltimore, MD USA; 5 Centre for Global Health and Quality, Georgetown University Medical Center, Washington, DC USA; 6 Division of HIV, ID and Global Medicine, University of California, San Francisco, Zuckerberg San Francisco General Hospital, San Francisco, CA USA; 7 Department of Epidemiology, Johns Hopkins School of Public Health, Baltimore, MD USA

**Keywords:** Patient satisfaction, Re-engagement, Lost-to-follow-up (LTFU), HIV, Zambia

## Abstract

Prognosis among those who are HIV infected has improved but long-term retention is challenging. Health systems may benefit from routinely measuring patient satisfaction which is a potential driver of engagement in HIV care, but it is not often measured in Africa, and Zambia in particular. This study aims to internally validate a patient satisfaction tool, assess satisfaction among patients previously lost-to-follow up (LTFU) from HIV care in Lusaka province and to measure association between patient satisfaction with their original clinic and re-engagement in HIV care. A cross-sectional assessment of satisfaction was conducted by tracing sampled patients drawn from public health facilities. Our findings suggest that satisfaction tool, previously validated in USA, exhibits high internal consistency for measuring patient satisfaction in the Zambian health system. Patient satisfaction with healthcare providers is associated with re-engagement in HIV care. Future interventions on patient-centred care are likely to optimize and support retention in care.

**Electronic supplementary material:**

The online version of this article (10.1007/s10461-019-02712-4) contains supplementary material, which is available to authorized users.

## Introduction

Globally, 36.9 million people were living with HIV/AIDS (PLWH) in 2017, the majority of who reside in low and middle-income countries [1]. Efforts to end AIDS as a public health threat by 2030 are underway throughout the most affected regions and globally, and in recent years antiretroviral therapy (ART) coverage across Sub-Saharan Africa has scaled up rapidly [2]. Zambia has an HIV prevalence of 12.8%, with nearly two-thirds of the estimated 1.2 million PLWH within the country receiving ART in 2016 [3].

Successes in treatment scale-up, however, have been threatened by challenges of retaining patients in long-term care. Data suggest that by 2 years post treatment, nearly 25% of ART patients are lost from care [4]. Identified factors driving low retention in care include health systems challenges (e.g. poor staff attitudes, congested clinics, long waiting times), psychosocial barriers (e.g. discrimination and stigma) and structural barriers (e.g. distance to health facilities, cost of public transportation) [5]. Patients have explicit expectations of health services and inadequate meeting of their needs may result in dissatisfaction [6].

Patient satisfaction is an important indicator of quality of healthcare services [7, 8]. However, satisfaction is also a complex construct that is difficult to measure. Patient reported satisfaction changes over time, and there is not a standard scale validated for use in Southern Africa [9]. To improve health services, health systems may benefit from routinely measuring patient satisfaction and considering it as a potential driver of retention in HIV care. Yet, despite emerging evidence from studies on patient-reported reasons for disengagement from HIV care, measurement of patient satisfaction for health services improvement remains rare in Africa, and Zambia in particular [10].

Measurement of patient satisfaction is one of the pillars of improving quality of health services and an integral part of understanding patients’ experiences within the healthcare system [11–13]. The objectives of this study were to internally validate a patient satisfaction tool, assess satisfaction among patients previously lost-to-follow up (LTFU) from HIV care in Lusaka province who had not returned to care at their original facility and to measure the association between patient satisfaction with their original clinic and re-engagement in care within two years. We hypothesized that patient satisfaction prior to LTFU positively impacts engagement in HIV care [14].

## Methods

### Study Design

Data collection was conducted in 2015 as part of a large study known as *Better Information for Health in Zambia* or the ‘BetterInfo study’ which was implemented in selected public health facilities supported by the Centre for Infectious Disease Research in Zambia (CIDRZ) in Lusaka, Southern, Eastern and Western Provinces. The main purpose of the BetterInfo study was to establish the health outcomes of patients who are lost-to follow-up (LTFU) through patient tracing which involved in-depth review of patients’ paper file and electronic medical records (EMR), phone communication and household follow up of patients in the community. The tracing process was conducted by CIDRZ-employed peer educators (tracers) and was guided by the Zambian Ministry of Health (MoH) tracing guidelines for LTFU patients [15]. A cross-sectional assessment of satisfaction was conducted among sampled adult LTFU patients drawn from a sub-sample of 13 Lusaka-based public health facilities supported by CIDRZ.

### Study Population and Sampling

The study population was comprised of HIV-positive adults (18 years or older) who had an HIV care and treatment visit between 1st August 2013 and 31st July 2015 at CIDRZ-supported public health facilities in Lusaka Province.

Identification of patients LTFU was determined by the Zambian National HIV Electronic database (‘SmartCare’). LTFU was defined as patients who had at least one HIV care visit between 1st August 2013 and 31st July 2015 and had no visit documented in SmartCare for 90 days since a missed appointment or 180 days since any recorded care visit [16]. As it was not possible for tracers to follow-up all LTFU patients to ascertain their re-engagement status, a multi-level, stratified sample of public health facilities and LTFU patients within each health facility was used for intensive tracing. We randomly selected 10% or approximately 150 LTFU patients at each facility for manual file review and active tracing; those who had a recent visit or deceased vital status in their paper record were excluded. Patients successfully contacted in-person were invited to participate in the study by trained tracers with experience working in ART clinics. Re-engagement in care at another clinic was ascertained through self-report during active tracing. Patients who were under 18 years at their last visit date, or who were unable to provide informed consent or give their responses in English, Nyanja, Bemba, Tonga or Lozi were excluded from the study.

### Study Procedures

Patients with unknown care status after medical record review were traced in the community. Once found, tracers encouraged patients to return to HIV care, conducted individual informed consent, and administered a tablet-based questionnaire including confirmation of current care status and satisfaction measures, along with other patient experience data.

### Study Measurements

We derived demographic characteristics of LTFU patients from the EMR. To measure patients’ satisfaction with their HIV care provider and care experience, we used a 9-item satisfaction scale adapted from the Adult Primary Care Questionnaire [17], previously validated in the United States. Briefly, the tool assessed various aspects of satisfaction with patient care using a 5-point Likert scale administered in two-steps: first whether the participant agreed, disagreed or felt neutral towards a statement, followed by assessment of the intensity of the agreement/disagreement (‘strongly disagree’ vs. ‘disagree’ and ‘strongly agree’ vs. ‘agree’). The nine items were: (i) provider spends enough time with patient; (ii) provider encourages patient to talk about health concerns; (iii) provider cares about patient as a person; (iv) provider listens carefully; (v) provider shows respect for what patient says; (vi) patient is confident of providers’ knowledge and skill; (vii) provider explains things in understandable way; (viii) provider explains reasons for any medical tests; and (ix) provider takes care of the patient. This tool was translated, put on a tablet computer and administered in the preferred language of the participant’s choice: Nyanja, Bemba, Tonga, or English. The outcome measure was re-engagement in HIV care, assessed through patient self-report.

### Statistical Analysis

Demographic characteristics of LTFU patients who completed the satisfaction survey were described and responses to the 9-item satisfaction scale individually reported [18]. To validate the satisfaction tool performance, we assessed both construct and criterion validity.

First, to assess construct validity, we estimated the number of underlying latent constructs represented by the scale through exploratory factor analysis (EFA). Using iterative principal factor estimation, we rotated the factors using oblique rotation, however based on the identification of only one factor, we then finalized the model using unrotated EFA. We assessed the internal consistency of the satisfaction scale across all 9-items of the tool using a Cronbach’s alpha. Following EFA, a total patient satisfaction score was calculated as a summation of responses to all 9-items on a 5-point Likert scale in which ‘Strongly Agree’ scored the highest at five and ‘Strongly Disagree’ scored the lowest as a one (range 9–45). We defined satisfaction as any score greater than 31.5 implying a mean score of 3.5 per question (indicating on average item responses were in agreement or above ‘neutral’).

Secondly, we assessed criterion validity through evaluating the association between satisfaction and re-engagement in care. Robust Poisson regression with clustering by facility was used to estimate the association between the patient satisfaction exposure (> 31.5 = satisfied; ≤ 31.5 not satisfied) and the outcome of re-engagement in care, adjusting for patient characteristics that we hypothesized a priori may be associated with both patient satisfaction and re-engagement, including age, gender, education and health facility-type [19]. Multiple imputation was used for missing satisfaction items [20, 21]. Participants missing more than 5 out of 9-scale items were excluded from the analysis (n = 5).

To assess the potential for misclassification of the exposure based on our satisfaction scale cut-point, particularly given that very strong dissatisfaction pertaining to any one item may strongly influence a patient’s overall satisfaction, we further assessed the degree to which those classified as ‘satisfied’ reported dissatisfaction to any of the items. A sensitivity analysis was performed using the robust Poisson regression models described above, which re-classified as ‘not satisfied’ any participants classified as ‘satisfied’ who reported that they were ‘strongly dissatisfied’ with any one of the nine items.

All data analyses were done using STATA version 15.0 (College Station, Texas).

## Results

### Demographic Characteristics

**Fig. 1:**
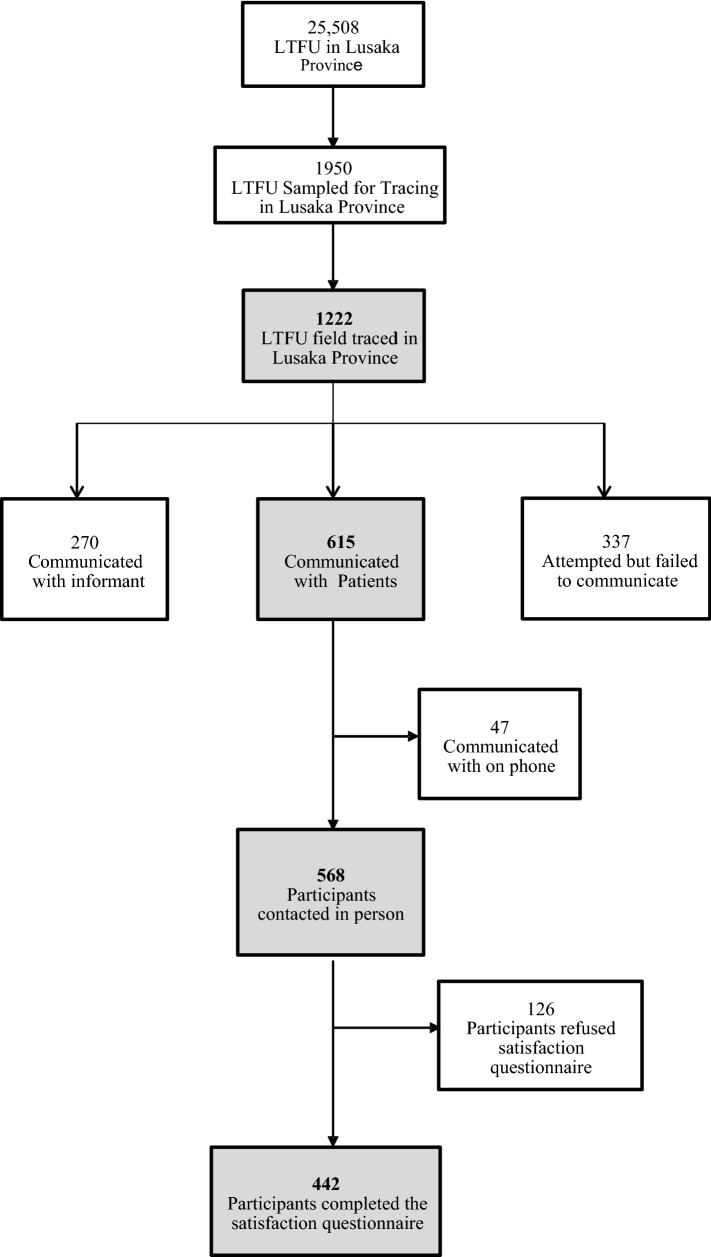
Flowchart of patient population

We traced 1222 LTFU patients in Lusaka Province, out of which 568 (46.5%) were found in person (an additional 47 were directly contacted via telephone). Overall, 442 (77.8%) of the patients traced in person completed the satisfaction survey, while 126 (22.2%) refused participation (Fig. 1). At time of in-person tracing, the median time since last known clinic visit was 1.6 years [IQR 1.2–2.1].

Among patients contacted in-person (n = 568), there were no significant differences between those who did and did not complete the satisfaction questionnaire except for facility type (11.5% vs. 8.7% in rural, 78.1% vs. 65.9% in urban and 10.4% versus 25.4% in hospital respectively, *p *< 0.01) (Table 1). The majority (n = 345, 78%) accessed HIV care and treatment from urban health facilities. Out of the 442 who completed the satisfaction questionnaire, 261 (59%) were female and the median age was 34.6 [IQR 29.7–39.9]. About half of the participants (52%) had attained upper basic or secondary school education and almost a third of participants (27%) had only attended lower basic education.

**Table 1:** Characteristics of traced lost to follow-up patients by completion of the satisfaction tool

Characteristic	All contacted (percentage of total)	Participants missing satisfaction data	Participants with satisfaction data	p value
	n (%)	n (%)	n (%)	
Gender				
Female	335 (59.0)	74 (58.7)	181 (41.0)	0.95
Male	233 (41.0)	52 (41.3)	261 (59.0)	
Age (year)				
18–30	184 (32.4)	42 (33.3)	142 (32.1)	0.41
31–40	251 (44.2)	49 (38.9)	202 (45.7)	
41–50	101 (17.8)	28 (22.2)	73 (16.5)	
50 +	32 (5.6)	7 (5.6)	25 (5.7)	
Education				
None	19 (3.3)	4 (3.2)	15 (3.4)	0.75
Lower-mid basic	146 (25.7)	27 (21.4)	119 (26.9)	
Upperbasic/secondary	298 (52.5)	70 (55.6)	228 (51.6)	
College/university	34 (6.0)	7 (5.5)	27 (6.1)	
Missing	71 (12.5)	18 (14.3)	53 (12.0)	
Facility type				
Rural	62 (10.9)	11 (8.7)	51 (11.5)	< 0.001
Urban	428 (75.4)	83 (65.9)	345 (78.1)	
Hospital	78 (13.7)	32 (25.4)	46 (10.4)	

### Factor Analysis and Internal Consistency of Satisfaction Scale Items

All the 9-scale items loaded to a single latent factor (minimum factor loading of 0.61 and a maximum of 0.76), which we deemed to be overall satisfaction (Table 2). Given the high loadings and low uniqueness of all nine items, all items were retained, including ‘provider spends enough time with me’, which had a uniqueness greater than 0.5, but with a high factor loading. The internal consistency across items was high with a Cronbach alpha score of 0.93 (α = 0.93). EFA results estimated that there was only one factor with an eigen value greater than 1.

**Table 2:** Internal consistency of satisfaction scale items

Variable	Factor 1	Uniqueness
Provider takes care of me	0.7075	0.4510
Provider explains the reasons for any medical tests	0.6985	0.4422
Provider explains things in a way that is understandable	0.7649	0.3908
I am confident of medical provider’s knowledge and skills	0.6821	0.4796
Medical providers show respect for what I say	0.7596	0.3933
Provider listens to me carefully	0.7515	0.3969
Provider cares about me as a person	0.7556	0.3840
Provider encourages me to talk about all my health concerns	0.7270	0.4404
Provider spends enough time with me	0.6143	0.5938

### Levels of Satisfaction with HIV Care Providers

**Fig. 2:**
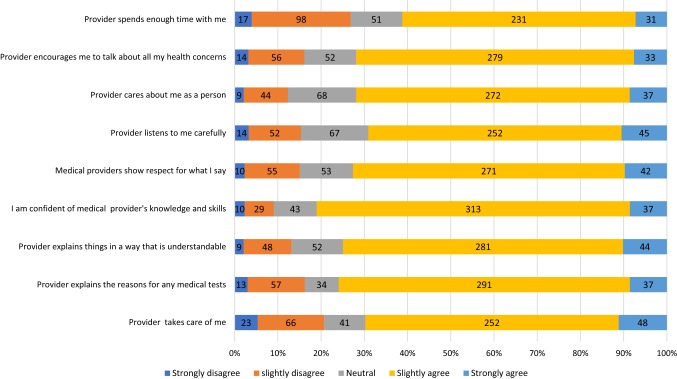
Level of satisfaction by satisfaction scale items. Bars represent the proportion of participants agreeing with each statement; numbers within the bars represent the absolute number of individuals providing each response

Figure 2 illustrates the levels of self-reported satisfaction of LTFU patients with their health care providers. In general, patients were satisfied with their healthcare providers. However, the items with the greatest proportion of patients disagreeing were ‘provider spends enough time with me’ (39%) and ‘provider listens to me carefully’ (31%).

**Fig. 3:**
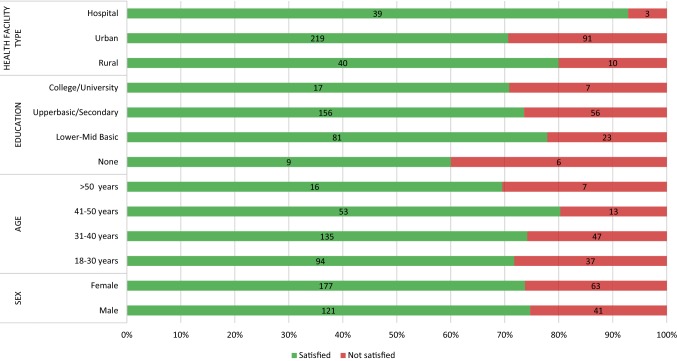
Satisfaction by patients demographic characteristics. Bars represent the proportion of participants satisfied according to patient characteristics; numbers within the bars represent the absolute number of individuals satisfied versus not satisfied

Overall, 298 patients (74.1%) were satisfied with their healthcare providers (scale score > 31.5). Differences were observed in overall satisfaction among patients according to health facility type (13.4% in rural, 74.4% in urban and 13.0% in hospital, *p *< 0.01), however there were no differences reported based on education (*p *= 0.65), age (*p *= 0.58) or sex (*p *= 0.83) (Fig. 3). Patients with no formal education most commonly expressed non-satisfaction (40%) while those who accessed HIV care and treatment services from a hospital most frequently expressed that they were satisfied (93%).

**Fig. 4:**
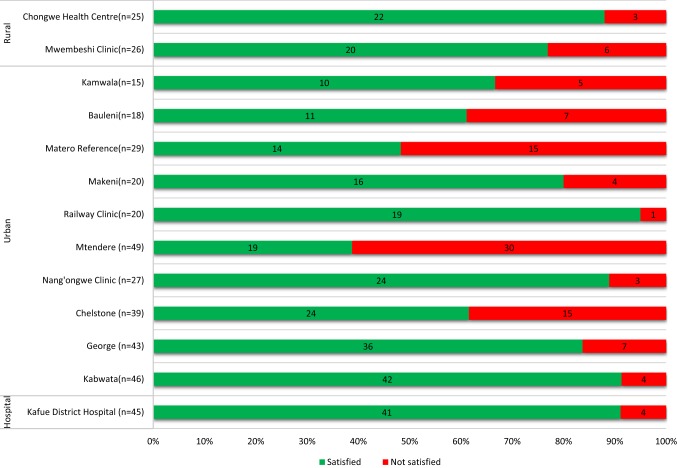
Satisfaction by health facility. Bars represent the proportion of participants satisfied by facility; numbers within the bars represent the absolute number of individuals satisfied versus not satisfied

There was, however, marked heterogeneity observed in satisfaction across health facilities (Fig. 4) regardless of urbanicity and type of facility.

Overall, 185 out of 437 (42.3%) patients were found to have returned to care. Among those returning to care, 98.1% re-engaged in their original health facility. In the Poisson regression model, patients who were satisfied with their HIV providers were significantly more likely to re-engage in care than those who were not satisfied (prevalence ratio [PR] 1.55, 95% CI 1.23, 1.94, p = 0.000). The association between satisfaction and re-engagement was maintained after adjusting for patient age, gender, health facility type and time since last visit (aPR 1.67, 95% CI 1.1.34, 2.07, p < 0.001). We assessed evidence of effect measure modification of the relationship between satisfaction and re-engagement across facility type; while there was preliminary evidence of an effect measure modification (strong relationships between satisfaction and re-engagement within urban and hospital facilities, but no relationship observed in rural areas), sample sizes for analysis by facility type were inadequate and estimates are reported for the full model alone.

Overall, 7% of participants who were classified as ‘satisfied’ expressed strong dissatisfaction to at least one item (breakdown by item provided in Online Appendix). In a sensitivity analysis reclassifying those who were strongly dissatisfied on any item to ‘not satisfied’, results of the robust Poisson regression remained largely unchanged (aPR 1.55, 95% CI 1.28, 1.87, p < 0.001).

## Discussion

This study assessed satisfaction among HIV patients who previously had an unknown vital and care engagement status in Lusaka province and its associations with re-engagement in care. Patients were generally very satisfied with their care, though there was heterogeneity across clinics. This could be consistent with the hypothesis that health facility factors drive patient experience [22]. Furthermore, these data suggest that satisfaction was related to re-engagement in care and thus may be an important measure to programmatically monitor as well as intervene on.

We found that the patient satisfaction scale previously validated in the US showed high internal consistency for measuring satisfaction in Zambia. Our findings suggested that the instrument measures a single latent construct which we referred to as ‘overall satisfaction’. Criterion validation was supported through the association between satisfaction with healthcare providers and engagement in care. Together these findings suggest the potential utility of this instrument in other Sub-Saharan Africa (SSA) settings.

Patient satisfaction has the potential to affect engagement in HIV care [18].This is consistent with a study conducted in Tanzania in which it was found that disrespectful and often abusive treatment by service providers following an absence from care was the leading factor of disengagement and reluctance to return [23]. In addition, in a study on understanding preferences for HIV care and treatment in Zambia, patients were willing to expend considerable time and effort as well as accept substantial inconvenience in order to access providers with a good attitude [24]. These findings highlight the critical need to improve provider–patient relationships in HIV care and treatment programs.

Overall, 74% of patients expressed satisfaction with healthcare providers. While patients perceived their healthcare providers to be clinically competent, aspects of HIV care delivery such as time spent with patients, taking care of patients and careful listening could be improved. Several other studies have found that long waiting times contribute to satisfaction and this may be compounded by the fact that patients spend insufficient time with healthcare providers and this makes it difficult for patients’ concerns to be heard [25, 26].

High levels of patient satisfaction (69%) were also reported in a facility-based Zambian study conducted among HIV and non-HIV patients where they found that facility characteristics such as management (government, non-governmental organisation or private), location (urban/peri-urban or rural) and facility type (hospital or health centre/health post) were important determinants of patient satisfaction [27]. However, it was only health facility type in our study, that predicted satisfaction. While our study reaffirms high levels of satisfaction reported in other studies, we specifically examined satisfaction among patients previously LTFU and expected to potentially see greater dissatisfaction with healthcare providers based on qualitative findings from the larger BetterInfo study which showed that bad attitudes of healthcare providers among other reasons, predicted disengagement from HIV care [28]. However, as hypothesized, we did observe that satisfaction was associated with re-engagement in care. This could imply that these patients were content and confident with the healthcare provision in the health facilities where they re-engaged. On the other hand, those who were not satisfied may have been influenced by individual factors (e.g. adherence, lack of time, side effects) or non-provider characteristics of the health facility, such as long waiting hours, lack of privacy, clinic operation hours, loss of patients’ files, and intensive adherence counseling for patients who missed their clinic visits [28].

The socio-demographic characteristics of LTFU patients did not strongly distinguish those who were satisfied with healthcare providers and those who were not. We did observe differences in satisfaction however by facility type, as well as heterogeneity by heath facility. Patients who were receiving care from urban facilities were less satisfied compared to those who were receiving care from rural health facilities and hospital. This could be explained by the high HIV prevalence in most urban areas of Zambia which has resulted in a rise in ART patient volumes without corresponding human resource increases to meet the demand in such urban-based health facilities [29]. In addition, dissatisfaction of urban-based patients in comparison to rural-based may be explained by differences in literacy levels. Patients in rural-based health facilities tend to have lower literacy levels and health knowledge, which may affect their perception of quality health service provision. Studies conducted in Ethiopia and Kenya on expectations and satisfaction of ART patients have shown higher satisfaction levels among those with lower literacy [30, 31].

Interpretation of findings should take into consideration study limitations. Although the broad sampling of LTFU patients across public health clinics in Lusaka is a strength of the study, results may not be generalizable to other settings, including to more rural settings in Zambia. Additionally, around half of patients randomly sampled for tracing were not found in person or refused the satisfaction questionnaire, potentially biasing the results if satisfaction was different among those who were and were not successfully traced. While we did not find any key differences in patients participating and refusing participation in the satisfaction survey, it is possible that our results over or underestimate patient satisfaction if there were underlying, unmeasured differences between these groups. Our measures relied on self-report and the data collection team was stationed in public health facilities, introducing themselves as those working in the sampled public health facilities in Lusaka province. This could have potentially introduced a social desireability bias as some LTFU patients might have over-reported socially desirable attitudes or under-reported socially undesirable attitudes of healthcare providers [32]. This has the possibility of overestimating the levels of satisfaction. But when we consider the general population, it is also possible that patient satisfaction would be underestimated as our sample consisted of LTFU patients. Another limitation is that we didn’t assess satisfaction with overall healthcare, taking into account facility characteristics. Instead our focus was on the healthcare providers. Thus, we may have missed some meaningful dimensions of dissatisfaction with healthcare not associated with the healthcare provider. Furthermore, there may be other factors associated with satisfaction and re-engagement that were not assessed leading to residual confounding. A key limitation is that of temporality. Ideally, satisfaction would be measured before LTFU and compared among those who were lost and not lost. As we only have cross-sectional data amongst those who were lost, we have assessed the relationship with re-engagement in care, but recognize that causality cannot be determined. Finally, although patients were asked to reflect on their satisfaction with services prior to disengagement, it is possible that some participants who had re-engaged in care responded based on their satisfaction with more recent services if they had re-engaged.

## Conclusion

We found that patient satisfaction with healthcare providers is associated with re-engagement in HIV care among patients LTFU. Measuring patient satisfaction may be an important element of improving retention in HIV care efforts and will likely become more important as HIV service delivery models expand [33]. These findings also offer encouragement in the midst of health system challenges (long waiting times, congestion in health facilities, staff shortages) that patients are largely satisfied with their healthcare providers. However, these data also reinforce the importance of interventions that improve patient experience at the health facility level as this may improve engagement in care. Further research is needed to better understand what can feasibly be done to improve patients’ experience in public health facilities and to understand how alternative delivery of care models may alter satisfaction.

## Electronic supplementary material

Below is the link to the electronic supplementary material.

Supplementary material 1 (DOCX 15 kb)
