# Supplementary material for: Patients’ Satisfaction with HIV Care Providers in Public Health Facilities in Lusaka: A Study of Patients who were Lost-to-Follow-Up from HIV Care and Treatment
Source: AIDS Behav. 2019 Oct 31;24(4):1151–60. doi: 10.1007/s10461-019-02712-4 (PMC7082366; doi:10.1007/s10461-019-02712-4)
Supplement: Supplementary file 1 — Supplementary material 1 (DOCX 15 kb) [file 10461_2019_2712_MOESM1_ESM.docx]

**Appendix 1: Distribution of satisfaction responses across the subset of participants classified as “satisfied” by item**. Bars represent the proportion of participants satisfied by item; numbers within the bars represent the absolute number of individuals.
